# Some Cases of Enuresis

**Published:** 1905-11

**Authors:** J. McF. Gaston

**Affiliations:** Atlanta, Ga.


### SOME CASES OF ENURESIS.

BY J. McF. GASTON, A.M., M.D., ATLANTA, GA.

### ENURESIS DIURNA ET NOCTURNA.

Having met with a very stubborn case of incontinence of urine soon after beginning the practice of medicine, eight years ago, and again very recently, it seemed to me the experience gained may be useful to others.

Incontinence of urine in children is a comparatively frequent disease, and gives a great deal of concern to parents, not to speak of the actual inconvenience to the patient and all in the house.

Incontinence in the young adult of both sexes is also very much more common than might be supposed, while the disease is of deeper origin and greater gravity.

We may also have incontinence of urine in the aged, giving rise to symptoms of grave import.

While incontinence of urine is a symptom rather than a disease, we must regard the trouble as one demanding attention to correct this abnormal function. The symptoms are rather common in many cases of cystitis, pyelitis, malposition of uretus, stone in the bladder, urethritis, or neuroses of various kinds.

No case of incontinence of urine should be passed over lightly, but should be investigated thoroughly as to its cause.

A very marked acidity of urine should be investigated likewise. The simple irritation of highly acid urine may give rise to frequent micturition, and at times to involuntary urination.

The history of many cases will be of great benefit in the explanation of a case of incontinence of urine, and should be carefully sought.

A child should be questioned as to the existence of any pain after urination, or whether the urine burns.

In the case of males a child may be suffering from the effects of phimosis, which may be accompanied by retention of urine, and later paralysis of bladder and incontinence of urine.

The reflex neuroses in children from the existence of a tight or elongated prepuce are many; also from such as clitoridean adhesions, worms and vulvitis.

CASE I.—C. J., aged 19. History: Disease of some duration. Was more severe in the latter part of 1893. He had been treated by several physicians and gave account of no relief by medicines.

Circumcision resorted to by me. Delayed urine and hemorrhage. Bromides and antiphlogistic treatment was used. Incontinence persisted after the healing of wound. Treatment by sounds for stricture gave some relief. Then was treated by electricity, using the faradic battery with a metallic electrode to feet and spinal column and a large brush on the perineum, penis and genito-urinary tract.

Baths in Ponce de Leon pond gave some partial relief.

Belladonna, ergot, rhubarb, bicarbonate potash, uva ursa, and the usual medicines, were only partially successful.

He could not hold urine, so that finally he was given a urinal day and night.

In the day he had no great difficulty for an hour or more, but could not go into company for fear of being unable to hold urine.

Improvement gradual after year or more, when he was at work, and he has not been heard from since, except once, when he was advised to have stricture treated by internal urethrotomy.

CASE II.—F. S. W., aged 18. Treated for incontinence of urine. He was apparently healthy otherwise. As a neurosis, it was treated by atropine in increasing doses. After the toxic effects were reached, he decreased dose.

Improvement from first dose. Completely cured after two weeks of treatment.

Saw him two years, or more, later and found him entirely well in this particular.

CASE III.—A. M., aged 10. Negro boy. Treated at surgical clinic of Southern Medical College. Neurosis. Treated by atropine. Cured.

CASE IV.—Carrie, negro woman, aged 23. Excessive venery probable cause.

Searched for stone in bladder with negative result.

Treated by demulcents and opiates.

Relief immediate, but no important or radical cure.

CASE V.—Dick C., aged 10. Negro boy. Nocturnal incontinence from habit.

Treated with a pill of ergotin, ½ gr., strychnine 20, extract belladonna, 1-64 gr. Cured.

CASE VI.—F. C., aged 12. White boy. Neurotic temperament. Masturbation suspected. Had close, elongated prepuce, dilated, and adhesion broken up. December 1899. Also given above pills, only temporary results.

But later return of nocturnal incontinence which became very annoying to parents, inasmuch as bed was soaking even to dripping through onto floor.

Admitted to Presbyterian Hospital in August, 1901. Circumcision performed. Nurses at hospital reported bed wet night after operation ; and parents reported same after his return home for a short time.

After healing of wound used a small bottle of medicine containing atropine sulphate in doses of 1 or 2 grs. three times daily. Patient was cured and so far no return of symptoms.

CASE VII.—E. H., aged 39. Married negro woman. Complained of inability to retain water during day. Acidity of urine and deficiency of bile led to treatment by calomel and soda with complete relief and cure.

CASE VIII.—E. G., aged 15. Complained of inability to hold water, and as the condition was of the same character as preceding case, she was put on calomel and soda, with cure.

CASE IX.—A. B., aged 76. Suffered with incontinence after severe attack of cystitis with pus in urine. Neuroses supposed to be primary cause. Use of atropine first and then of pill mentioned gave relief.

There occurred spasmodic involuntary micturition during the effort to reach a vessel, or at times before fairly up in bed, when a feeling of urgent urination had come on. This seemed to be in the nerve center controlling the sphincter of the bladder, and more in the great preponderance of power still residing in the detrusor muscles than in the actual paralysis of sphincter. There was need of some tonic effect upon the sphincter, but also a co-ordination of the nerves which supplied the detrusor and sphincter nerves.

CONCLUSIONS.

1. Atropine sulphate is a specific for neurotic enuresis, with other things equal.

2. That all the cases required judicious discrimination as to cause.

3. That great caution should be used in the use of atropine.

4. That circumcision is indicated not only in phimosis, but elongated loose prepuce, to cure enuresis.

5. That hypertrophy of the prostate, urinary calculi, and hyperacidity should be treated for relief of enuresis.

6. Many cases may not yield to medical means, but may finally be treated surgically.

